# Prevalence of congenital heart disease according to the echocardiography findings in 8145 neonates, multicenter study in southern Iran

**DOI:** 10.1002/hsr2.1178

**Published:** 2023-04-04

**Authors:** Seyedeh Yasamin Parvar, Rezvan Ghaderpanah, Amir Naghshzan

**Affiliations:** ^1^ Student research committee Shiraz University of medical sciences Shiraz Iran; ^2^ Cardiovascular and Neonatology research center Shiraz University of Medical Sciences Shiraz Iran

**Keywords:** congenital heart disease, echocardiography, epidemiology, Iran, pediatrics

## Abstract

**Introduction:**

Congenital heart disease (CHD), the lethal congenital anomaly in newborns, is multifactorial, with environmental and genetic factors contributing to its occurrence. Although some studies on the prevalence of CHD have been conducted throughout the country, this large‐scale study aims to provide information on the prevalence of various types of CHDs in newborns according to the echocardiography findings.

**Patients and Methods:**

Over 3 years, 8145 neonates with suspected CHD who underwent echocardiography by a trained pediatric cardiologist were included in this multicenter, cross‐sectional observational study. CHD was categorized into two major groups; cyanotic and acyanotic heart disease. The SPSS version 22 software was used to analyze the data with a significance level set at 0.05.

**Results:**

Of 8145 neonates who were referred to our centers with CHD symptoms, 6307 were indicated for echocardiography. The mean age of the studied population was 8.5 ± 9.3 days and the male‐to‐female ratio was 2.6, especially in the arterial septal defect (ASD), ventricular septal defect (VSD), and patent ductus arteriosus (PDA) groups. 77.2% of patients had acyanotic heart disease (100 in 1000 neonates) with ASD as the most common one and 9% were diagnosed with cyanotic heart disease (11 in 1000 neonates) with transposition of the great arteries as the most common form and the aortopulmonary window was the rarest form.

**Conclusion:**

This large prospective, multicenter screening study reported arterial septal defect (85%) and patents with ductus arteriosus (32%) as the most frequent type of CHD. Moreover, the prevalence of male patients was significantly higher. This information would be helpful for health policy makers, stakeholders, and general practitioners in regions where there are no trained pediatric cardiologist fellowships and limited access to echocardiography devices for better management of CHD.

## 1 INTRODUCTION

Congenital heart disease (CHD) is a group of abnormalities in the heart structure or cardiovascular function occurring from birth, even if diagnosed later.^1^ These cardiac defects range from minor lesions with no clinical manifestations to potentially fatal conditions.^2^ The top five most common acyanotic CHDs are the followings: arterial septal defect (ASD); pulmonary stenosis (PS); ventricular septal defect (VSD), and patent ductus arteriosus (PDA), and tetralogy of Fallot (TOF).^3^

Despite numerous published studies, the exact causes of CHD have not yet been fully discovered. CHD appears to be a multifactorial disease caused by environmental and genetic factors.^4^ Environmental factors include a history of maternal illness during pregnancy, a positive familial history, the number of pregnancies, maternal hypertension, and medication exposure during pregnancy.^5^ Moreover, mortality has increased over the past decades due to a lack of appropriate diagnostic and treatment facilities in low‐ and middle‐income countries.^6^

CHD is newborn's most common lethal congenital disorder, accounting for roughly one‐third of all congenital birth defects.^2^, ^7^ At least 1.5 million children are born each year with CHD,^8^ and it is a great emerging problem in pediatric global health. Although we may not be able to change the prevalence of CHDs, resources and screening, as well as effective interventions, are required to improve patients’ survival and prognosis.^9^

All types of CHDs may severely reduce the quality of life of all family members, impose high costs on the health care system, necessitate adulthood follow‐ups, repeated surgical procedures.^1^ In our country, several studies have reported a prevalence between 4 and 8 per 1000 live births.^10^ The systematic review conducted in 2019 found a broad range of CHD prevalence in Iran which necessitates further extensive epidemiological studies in our region. The prevalence of CHD varies greatly among populations and geographic locations. Therefore, it is essential to study the epidemiology of CHD in different countries and populations for understanding its underlying causes, risk factors, and potential preventive measures. As compared to earlier published articles, there are a number of novel aspects to the present study. First, the sample size of more than 8000 inpatient neonates is relatively large, providing a more robust representation of the population under study, increasing the statistical power of the study, and enabling researchers to detect smaller differences and associations between factors. Secondly, our study is unique in terms of its focus on Iran, a country that has not been extensively studied regarding CHD epidemiology. As such, this study can provide important insights into the prevalence and distribution of different subtypes of CHD in our region, which can aid the identification of high‐risk groups and inform strategies for the early detection and management of CHD in neonates. Finally, this study can provide valuable information on the clinical and demographic characteristics of neonates with CHD in Iran, including the types of defects and associated comorbidities. This information can help clinicians and researchers better understand the clinical course of CHD and its impact on neonatal health not only in Iran but also in other neighborhood countries.

The current study aims to assess the epidemiology and prevalence of common CHD subtypes in a huge group of neonates referred to our hospitals in southern Iran. The study province comprises central facilitated cardiology departments in Iran, and each year, many CHD patients from all over the country seek treatment at our hospitals.

## 2 PATIENTS AND METHODS

This cross‐sectional, retrospective, and multicenter study was conducted between March 2018 and October 2021 using hospital records from four major hospitals affiliated with Shiraz University of Medical Sciences. After obtaining approval from the ethics review committee, the study was conducted in accordance with the Declaration of Helsinki and the STORB checklist.

Inclusion criteria consist of all inpatient neonates (aged under 30 days) admitted to the pediatric cardiology department and neonatal intensive care unit (NICU) or referred for cardiology consultation from other wards in our hospitals who were suspected of having common types of CHD (patients with cyanosis, syndromal disorders, heart murmurs, failure to thrive, respiratory distress, or arrhythmogenic cardiac disorders) Exclusion criteria were patients with unstable condition, not indicated to echocardiography, dissatisfaction of their parents for inclusion to the study, patients with incomplete or duplicate datasheets, aged older than 30 days with functional murmur, and features not representative of CHDs. CHD cases were divided into two categories:

- Cyanotic heart diseases consist of TOF, pulmonary atresia, double outlet right ventricle (DoRV), transposition of great arteries (TGA), single ventricle, pulmonary vein anomalies, truncus arteriosus, atrioventricular septal defects (AVSD), aortopulmonary window (AP Window).
- Acyanotic heart diseases consist of ASD, VSD, PDA, pulmonary stenosis (PS), coarctation of the aorta (CoA), Aortic stenosis (AS), Right and left ventricular hypertrophy (RVH and LVH), and mitral valve prolapse (MVP).

During the study period, 8145 live‐born newborns were suspected of having CHD and were subjected to echocardiography. Data of the current study were obtained from patients’ records and online discharge sheets filled out by medical students and cardiology attendings at the SUMS Clinical database (http://ped.sums.ac.ir). Echocardiography reports and demographic information such as age, gender, and admission date were recorded in the excel sheet for further coding and data analysis.

### 2.1 Echocardiography

A board‐certified cardiologist performed a thorough history and physical examination of the patients. In the event of any suspicion of CHD, echocardiography was performed in accordance with standard transthoracic echocardiography guidelines.^11^ The M‐mode, two‐dimensional, and color Doppler parameters were obtained using a Samsung HS70 or a GE Vivid 3 echocardiography machine and recorded in the university's database system. Two‐dimensional and color doppler imaging from standard views were part of the examination protocol (Parasternal Long Axis, Parasternal Short Axis, Apical 4 Chamber, Subxiphoid (Subcostal), and apical). Sedation was not routinely used, and most cases were performed while the patient was sleeping in the echocardiography room. Furthermore, the echocardiography data of Complex CHD were reviewed and confirmed by second‐hand pediatric cardiologists before entering into the database system.

Indications of echocardiography:

1. Cyanosis and low O2 saturation early after birth.
2. O2 saturation less than 95% 24 h after delivery.
3. Pathologic cardiac murmurs in routine physical examination.
4. High‐risk neonates (history of diabetic Mellitus in mother, an autoimmune disease in mother, maternal hypertension, group x medications exposure during pregnancy.)
5. History of complex CHD in other siblings.
6. Neonates with antenatally diagnosed with CHD.

### 2.2 Statistical analysis

The data is described using mean, standard deviation, frequency, and percent. The statistical package for social sciences (SPSS Inc.) version 22.0 was used for all statistical analyses. The *χ* ^2^ test was performed to compare two genders in each disease, with the significance level set at 0.05. We also used two proportion test to compare the frequency between genders with the significance level of 0.05. Normality of data was assessed using Kolmogorov–Smirnov test.

## 3 RESULTS

**Figure 1:**
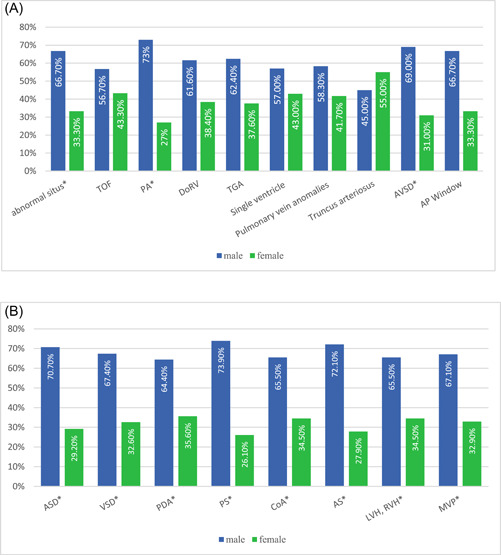
(A) The frequency of cyanotic CHDs among genders based on proportion test. (B) The frequency of acyanotic CHDs among genders based on proportion test. *p < 0.05. **Percent is represented based on total patients in each category. ASD, atrial septal defect; AS, aortic stenosis; CoA, coarctation of the aorta; LVH, left ventricular hypertrophy; MVP, mitral valve prolapse; PDA, patent ductus arteriosus; PS, pulmonary stenosis; RVH, right ventricular hypertrophy; VSD, ventricular septal defect.

After thorough history taking and physical examination of 8147 neonates with suspected CHD, 6307 (77.4%) cases were indicated for echocardiography. The average age of the studied population (those who underwent echocardiography) was 8.5 ± 9.3 days, ranging from 0 to 30 days. Overall, the prevalence of CHD was significantly higher in male neonates, resulting in a male‐to‐female ratio of 2.6 (*p* < 0.001). According to the *χ* ^2^ test, the prevalence of male patients was significantly higher in cyanotic patients with single‐ventricle, TGA, DoRV, and TOF disease and in the acyanotic patients with MPV, RVH, LVH, PDA, and VSD disease (*p* < 0.05). Whereas the proportion of male‐to‐female was significant in all types of acyanotic and in abnormal situs, PA, and AVSD among cyanotic types as shown in Figure 1A,B. Descriptive and analytic statistics of the extracted information are shown in Tables 1 (cyanotic CHD) and 2 (acyanotic CHD).

**Table 1:** Echocardiographic profile of cyanotic congenital heart disease.

Types of cyanotic congenital heart disease		Total frequency *N* (%)	Male *N* (%)*	Female *N* (%)*	*p* Value**	Mean age ± SD (days)
Abnormal situs	Total	63 (10)	42 (1.1)	21 (1.1)	0.48	8.48 ± 8.66
	Dextrocardia	37 (58.7)				
	Situs inversus	27 (42)				
	Situs ambiguus	21 (33.3)				
	Mesocardia	6 (9)				
Tetralogy of Fallot (TOF)		120 (19)	68 (1.5)	52 (2.8)	<0.001	7.08 ± 7.50
Pulmonary atresia (PA)	Total	100 (16)	73 (1.6)	27 (1.4)	0.94	7.84 ± 9.61
	PA‐IVS	86 (86)				
	PA + VSD	14 (14)				
Double outlet right ventricle (DoRV)		73 (11.7)	45 (0.98)	28 (1.5)	0.094	9.44 ± 7.91
Transposition of Great arteries (TGA)	Total	149 (24)	93 (2)	56 (3)	0.029	7.37 ± 7.31
	TGA‐IVS	74 (49.6)				
	TGA + VSD	63 (42.2)				
Single ventricle	Total	128 (20)	73 (1.6)	55 (2.9)	<0.001	6.46 ± 7.38
	Mitral Atresia	36 (21.3)				
	Hypoplastic LV	43 (25.4)				
	Tricuspid atresia	36 (21.3)				
	Hypoplastic RV	54 (31.9)				
	Large VSD	0				
Pulmonary vein anomalies	Total	127 (20)	74 (1.6)	53 (2.9)	0.93	5.98 ± 7.07
	Cortriatrium	2 (0.1)				
	PAPVC	50 (39.3)				
	TAPVC	23 (18.1)				
Truncus arteriosus		20 (3)	9 (0.2)	11 (0.59)	0.23	9.65 ± 9.40
Atrioventricular septal defects (AVSD)		42 (6)	29 (0.65)	13 (0.7)	0.86	9.81 ± 8.78
Aortopulmonary window (AP window)		3 (0.4)	2 (0.04)	1 (0.05)	1	7.00 ± 6.24

* % is represented within genders.

** *p* value is represented based on gender and *χ* ^2^ test.

Six thousand two hundred and ninety (77.22%) of 6307 neonates who underwent echocardiography had acyanotic heart disease (100 in 1000 neonates) of which, ASD was the most common one, accounting for 65.9% (85 in 1000 neonates) especially PFO and small ASD second types. PDA was the second most common acyanotic heart disease comprising for 32.5%, followed by VSD (15.4%). CoA was the least common defect in the acyanotic category (7 in 1000 neonates). Only 731 (8.97%) participants diagnosed with cyanotic heart disease (11 in 1000 neonates). TGA was the most common cyanotic CHD, accounting for 1.8% (2 in 1000 neonates), followed by TOF, the presence of a single ventricle (primarily in patients with hypoplastic right ventricle), and pulmonary vein anomalies respectively. On the other hand, truncus arteriosus and AP window were the least common types of CHD, respectively (0.04 in 1000 neonates).

**Table 2:** Echocardiographic profile of acyanotic congenital heart disease.

Type of acyanotic congenital heart disease		Total frequency *N* (%)	Male *N* (%)*	Female *N* (%)*	*p* Value**	Mean age ± SD (days)
Atrial septal defect (ASD)	Total	5369 (85)	3798 (85)	1575 (84.8)	0.64	8.68 ± 9.27
	PFO	2914 (54)				
	Secundum	2266 (42)				
	primum	47 (0.8)				
	Sinus venosus	4 (0.07)				
	Common Atrium	17 (0.3)				
Ventricular septal defect (VSD)	Total	968 (15.4)	653 (14.6)	316 (17)	0.017	9.25 ± 8.90
	Perimembranous	494 (51)				
	Inlet	6 (0.6)				
	Muscular and Apical	177 (18)				
	SubAortic and subpulmonic	62 (6.4)				
Patent ductus arteriosus (PDA)		2039 (32.5)	1314 (29.5)	725 (39)	<0.001	5.32 ± 7.47
Pulmonary stenosis (PS)	Total	448 (7)	331 (7.4)	117 (6.3)	0.11	10.43 ± 9.60
	Mild	85 (18)				
	Moderate	33 (7)				
	Severe	43 (9.5)				
Coarctation of the aorta (CoA)	Total	168 (2)	110 (2.4)	58 (3.1)	0.14	9.18 ± 8.27
	Discrete	140 (83)				
	Long segment	33 (19.6)				
	Interrupted Arch	22 (13)				
	Hypoplastic arch	77 (45)				
Aortic stenosis (AS)	Total	270 (4)	194 (4.3)	75 (4)	0.63	6.52 ± 8.45
	Mild	24 (8)				
	Moderate	12 (4)				
	Severe	20 (7)				
Left and right ventricular hypertrophy (LVH and RVH)		753 (12)	493 (11)	260 (14)	<0.001	7.55 ± 8.48
Mitral valve prolapse (MVP)		709 (11)	476 (10.6)	233 (12.5)	0.032	5.77 ± 8.10

* % is represented within genders.

** *p* value is represented based on gender and *χ* ^2^ test.

## 4 DISCUSSION

In the present population‐based study we identified 6307 patients with CHD among 8145 live neonates suspected to CHD. Our results revealed that the most common cyanotic defects were TGA (2 in 1000 neonates), TOF, and single ventricle, whereas the most common acyanotic defects were ASD (85 in 1000 neonates), followed by PDA and VSD. The study also showed a significantly higher prevalence of CHD among male neonates, with a male‐to‐female ratio of 2.6. This is consistent with previous studies, which have suggested a higher incidence of CHD among male neonates.^12^ It has been found differences in the prevalence of CHD by type, with acyanotic heart diseases being more common than cyanotic heart diseases. The results of this research offer significant epidemiological insights regarding the occurrence and categories of CHD among newborns in Iran. The high prevalence of CHD among neonates highlights the need for effective screening programs and early detection strategies to ensure timely and appropriate care.

The prevalence of congenital heart defects varies significantly across countries. The prevalence of CHD in Iranians ranges from 4.2 to 8.6 per 1000 live births.^13^, ^14^ Rahim et al. examined 3061 patients in western and southern Iran over 9 years and found that ASD was the most common type of congenital anomaly which was in line with our findings. Another study by Nikyar et al. conducted over 2 years reached the same conclusion.^14^, ^15^ In a recent article published in China, ASD was the most common CHD among all participants.^16^ In contrast to our study, VSD was reported as the most common CHD in some other studies.^15^, ^17^, ^18^ According to a study on 177 neonates, VSD was the most common type of congenital defect (44%), followed by ASD (21%).^13^ VSD has also been reported as the most common CHD in China and Japan.^19^, ^20^

In contrast to most published studies, in which ventricular defects (ASD or VSD) are the most common types of CHD, our results showed PDA to be the second most common type of congenital anomaly (32%) among all patients. Our findings revealed a higher incidence of PDA than in previous studies.^13^, ^21^ A study by Kafian et al. that examined 300 neonates reported a PDA prevalence of (12.8%) as the second most common type of CHD.^21^

Gender as a neonatal factor has been reported to have a significant effect on the type and presence of CHD so it can be considered a risk factor.^22^, ^23^ Our results show that the ratio of male to female neonates is 2.6, which means that CHD is more prevalent in male infants. The prevalence of male patients ranged from 45% to 73% of all conditions in the present study. The highest male propensity was found in neonates with PA and PS among cyanotics and acyanotic respectively. Male sex was significantly associated with TGA, TOF, and having single ventricle in cyanotic individuals and VSD and PDA in acyanotic individuals. These results are consistent with the findings of other studies.^14^, ^24^, ^25^ According to a study by Nikyar et al. they revealed that the incidence of CHD was significantly higher in male neonates (9.96 per 1000 live births compared with 7.34 in females).^14^ In another article, Wu et al. examined the worldwide incidence of CHD over a 20‐year period; the rate of CHD was higher in male neonates than in females (19.1 vs. 16.6 per 1000).^25^ In addition, two studies conducted in China and Germany reported that there was a male preponderance among patients with severe CHD.^20^, ^26^

On the other hand, when considering gender, our results contradict some studies showing female preponderance in congenital heart defects such as VSD, PDA, and ASD. For example, Amel et al.,^27^ who studied 3714 newborns from the south of Iran, found that a higher proportion of female infants suffered from VSD, PDA, ASD, and TOF. Another article showed a slight preponderance of females in the prevalence of CHD (VSD and PDA) on 2067 patients conducted by Ishikawa et al in China.^19^ However, the incidence of TOF was higher in male infants in the earlier study, which is consistent with the results of the current study. These differences between studies may suggest that CHD is multifactorial and that maternal and environmental factors vary widely from study to study. It is also worth noting that according to the National Organization for Civil Registration of Iran, the number of male newborns (139027) was significantly higher than that of female newborns (130720) during the study period.^28^

The current study's findings on the prevalence and types of CHD among neonates might have important impacts on healthcare providers, influencing targeted strategies to enhance the timely and appropriate care of this patient population. For instance, these findings can inform the development of screening programs that target high‐risk neonates for early detection and management of CHD, through initiatives such as encouraging early prenatal care and screening. Such programs can help to reduce mortality rates and improve long‐term health outcomes. In addition, the findings of this epidemiological study can inform the allocation of healthcare resources, including medical staff, equipment, and funding, to NICUs and pediatric cardiology centers, thereby ensuring that these facilities are adequately equipped to meet the specific needs of neonates with CHD. Furthermore, these findings can assist pediatricians and general physicians in better understanding the clinical course of CHD, including the types of abnormalities and associated comorbidities.^29^

Finally, the large study population of neonates is the main strength of this clinical survey. As a result, our findings can accurately represent the newborn population in southern Iran. Furthermore, we broadened this study's scope by looking at various cyanotic and acyanotic CHD abnormalities. All neonates in this study were subjected to an echocardiographic examination without being sedated. The absence of sedation was a double‐edged sword. On the one hand, there was no concern about the medication's side effects, which made it easier for the mothers to participate in the study. On the other hand, because echocardiography was usually performed during the newborns’ sleeping time, this was one of the reasons for its difficulty. Furthermore, our centers are well‐equipped, and board‐certified professionals perform echocardiography.

## 5 LIMITATIONS

Our study, like any other studies, had some limitations. First, we could not obtain the number of critically ill neonates who died during resuscitation before undergoing echocardiogram. Considering that most of our patients referred to us for diagnosis and treatment were neonates, asymptomatic patients or patients with minor abnormalities, such as tiny VSDs, were underrepresented. Second, our findings may be masked by the higher proportion of males born during the study period. Third, environmental factors were not included in our database. There is no uniform system for collecting CHDs data globally, so more detailed epidemiologic studies are required to determine the epidemiological patterns and risk factors in each region.

## 6 CONCLUSION

This large prospective, multicenter screening study investigates the prevalence of CHD in the southern part of the country. The most frequent types of CHD were arterial septal defect (85%) and patents with ductus arteriosus (32%). Inconsistent with other studies conducted in Iran, our study reported that acyanotic heart diseases were the most common forms of CHD among neonates. Moreover, the prevalence of male patients was significantly higher. This study provides valuable information about the prevalence and types of CHD among neonates in Iran. By taking into account the unique characteristics of this population, policymakers and healthcare providers can develop effective interventions that reduce the burden of CHD and improve the overall health outcomes of neonates with CHD. Further research is needed to better understand the underlying risk factors and causes of CHD among neonates in Iran.

## AUTHOR CONTRIBUTIONS

**Seyedeh Yasamin Parvar**: Conceptualization; data curation; investigation; resources; writing—original draft; writing—review and editing. **Rezvan Ghaderpanah**: Formal analysis; writing—original draft; writing—review and editing. **Amir Naghshzan**: Conceptualization; data curation; investigation; supervision.

## CONFLICT OF INTEREST STATEMENT

The authors declare no conflict of interest.

## ETHICS STATEMENT

The manuscript has been approved by all authors and has never been published or under the consideration for publication elsewhere. We confirm that all figures and tables are original and created by authors. We guarantee that all authors listed on the title page have read the manuscript and attest to the validity and legitimacy of the data. We would also like to undertake that we have read the plagiarism policy and submitted the article with complete responsibility. The Medical Ethics Committee of Shiraz University of Medical Sciences according to the declaration of Helsinki (IR. SUMS. REC.1399.1338).

## TRANSPARENCY STATEMENT

The lead author Amir Naghshzan affirms that this manuscript is an honest, accurate, and transparent account of the study being reported; that no important aspects of the study have been omitted; and that any discrepancies from the study as planned (and, if relevant, registered) have been explained.

## Data Availability

The data supporting the present study's findings are available on request from the corresponding author. However, they are not publicly available due to privacy and ethical restrictions.
